# Association between dietary patterns and anemia in older adults: the 2015 China adults chronic diseases and nutrition surveillance

**DOI:** 10.1186/s12889-025-22199-0

**Published:** 2025-03-19

**Authors:** Pengfei Wang, Qiya Guo, Xue Cheng, Wen Zhao, Hongyun Fang, Lahong Ju, Xiaoli Xu, Xiaoqi Wei, Weiyi Gong, Lei Hua, Jiaxi Li, Xingxing Wu, Li He

**Affiliations:** 1 National Institute for Nutrition and Health, Chinese Center for Disease Control and Prevention, Beijing100050, China; 2 Peking Union Medical College Hospital, Beijing100050, China

**Keywords:** Dietary patterns, Anemia, Elderly adults

## Abstract

**Background:**

Anemia is a condition that has been affected 1.92 billion people worldwide in 2021, leading physical decline, functional limitation and cognitive impairment. However, there are currently fewer studies focusing on the relationship between anemia and dietary patterns in older adults. This study aimed to analysis the dietary patterns in older adults aged 60 and above in China and their association with anemia.

**Methods:**

The data was obtained from the 2015 Chinese Adults Chronic Diseases and Nutrition Surveillance (2015 CACDNS), dietary information was collected using the food frequency method within the past year, exploratory factor analysis was used to extract dietary patterns, and logistic regression was used to analyze the relationship between dietary patterns and anemia.

**Results:**

A total of 48,955 elderly people were included, and the number of anemia patients was 4,417 (9.02%). Four dietary patterns were categorized by the exploratory factor analysis, two dietary patterns have been found to have a statistically significant relationship with the prevalence of anemia. Compared to the first quintile, the fifth quintile of dietary pattern 2 (DP2), characterized by high intake of rice and flour, fresh vegetables, livestock and poultry meat, aquatic products, was associated with higher prevalence of anemia in older adults (OR = 1.412, 95%CI: 1.273–1.567, *P* < 0.0001), and the trend test results showed that score of this dietary pattern was associated with higher prevalence of anemia (p for trend < 0.0001). Compared to the first quintile, Dietary Pattern 4 (DP4), rich in fungi and algae, fried dough products, other grains, various beans, and rice and flour, was linked to lower prevalence of anemia of the fifth quintile (OR = 0.768, 95% CI: 0.674–0.874, *P* < 0.0001). And DP4 score was associated with lower prevalence of anemia (*P* for trend < 0.0001).

**Conclusions:**

There were differences in dietary patterns among elderly people over 60 in China, and the prevalence of anemia in older adults was related to DP2, and DP4.

## Background

Anemia is a disease characterized by a decrease in hemoglobin or red blood cells, according to the World Health Organization (WHO) criteria, anemia is defined as a hemoglobin concentration < 130 g/L in men aged 15 years and above, and < 120 g/L in non-pregnant women aged 15 years and above [1]. Physical decline, functional limitations, and cognitive impairment are all associated with anemia. Additionally, anemia increases the risk of hospitalization, which may result from the exacerbation of existing health conditions or falls. Furthermore, it heightens the likelihood of being admitted to a nursing home, often due to a decline in functional abilities. The risk of mortality is also elevated, particularly when anemia is accompanied by other serious conditions such as heart failure, renal insufficiency, hypertension, or diabetes [2]. Anemia is also considered a risk factor for Heart Failure (HF), due to long-term chronic anemia can increase preload and decrease afterload. This can increase cardiac output, leading to undesirable left ventricular hypertrophy and left heart enlargement. Anemia may also exacerbate myocardial ischemia and lead to the progression of Coronary Artery Disease (CAD) due to reduced oxygen supply (or increased demand). And anemia is one of the independent risk factors for new-onset atrial fibrillation [3].

In 2021, the global prevalence of anemia across all ages was 24.3%, corresponding to 1.92 billion prevalent cases. This reflects a decrease from the 28.2% prevalence observed in 1990. Notable variations in the prevalence of anemia are observed across countries. Mali, Zambia, and Togo are the countries with the heaviest burden of anemia, with prevalence rates all above 50%, while Iceland, Norway, and Monaco are with the lightest burden, with prevalence rates all below 5% [4, 5]. The differences in the burden of anemia across countries may be related to dietary variations. The results about causes of anemia showed that dietary iron deficiency was the leading cause of anemia in most demographics [4]. And a research in Norway, one of the countries with the lightest anemia burden, found that the median intake of most nutrients met the Estimated Average Requirements of the 2012 Nordic Nutrition Recommendations. Participants in Norway contained significantly more iron and vitamin B12 [6].

In China, the overall prevalence of anemia among adults is 8.3%, with a prevalence rate of 5.9% for males and 10.8% for females. The prevalence rate among urban residents is 7.7%, while the prevalence rate among rural residents is 9.0% [7]. Furthermore, the prevalence of anemia increases steeply with age. The prevalence was less than 15% among individuals aged 60–64 years, while it exceeded 33.3% among those aged 80 years and above [8].

Numerous studies have explored the relationship between nutrients and anemia. For instance, heme iron serves as a critical component of hemoglobin [9]. Folate, an essential coenzyme for DNA synthesis, plays a pivotal role in erythropoiesis; its deficiency can inhibit the proliferation and maturation of red blood cells, leading to megaloblastic anemia [10]. Additionally, certain nutrients exhibit synergistic or antagonistic effects on iron metabolism: vitamin C enhances the absorption of non-heme iron, while other dietary components (e.g., calcium, phytates, polyphenols) may impair its bioavailability [11]. Investigating nutrient interactions is therefore vital for understanding and addressing anemia in the elderly population.

As individuals age, the functional decline of various organs and physiological systems becomes increasingly evident. In older adults, this manifests as diminished bone marrow regenerative capacity, which hinders erythropoiesis [12]. Thymic involution and structural atrophy reduce peripheral blood T-lymphocyte counts and immune function. Chronic inflammation, linked to immune dysfunction, and polypharmacy further disrupt iron metabolism and red blood cell production [13, 14]. Declines in chewing, swallowing, digestion, and nutrient absorption capabilities impair the ingestion, digestion and absorption of nutrients, reducing the bioavailability of critical nutrients such as iron, vitamin B12, and folate [15]. These age-related physiological changes collectively predispose older adults to a higher risk of anemia.

The relationship between food and disease can be truly and comprehensively reflected in dietary patterns. But at present, the majority of research on the relationship between dietary patterns and anemia has been conducted with pregnant women and children, with fewer studies focusing on older adults population [16–18]. Therefore, in the present study, we sought to analyze the dietary patterns of elderly individuals aged 60 and above in China, and further analyzed the potential relationship between dietary patterns and anemia in older adults. The study aimed to provide a scientific basis for preventing anemia in older adults from the perspective of dietary nutrition, based on the data of the 2015 CACDNS.

## Methods

### Study design and participants

Data for this study were obtained from the 2015 CACDNS. A complex, multistage, stratified cluster random sampling method was used to create a representative sample of 31 provinces, autonomous regions and municipalities in the mainland of China, including a total of 302 survey sites that were randomly selected. The initial stage of the study entailed the random selection of three communes/subdistricts from each survey site. Secondly, two communities or villages were randomly selected from each township or subdistrict. In the third stage of the process, one villager or residential group comprising a minimum of 60 households was randomly selected from each community or village. In the fourth stage, forty-five households were selected as survey households, and 20 households were selected as dietary survey households in each survey site, with a minimum of 612 residents aged 18 years and above in each survey site.

This study selected participants based on the following inclusion and exclusion criteria.

Inclusion criteria: Participants were required to have complete datasets, including:

1. Age ≥ 60 years old,
2. Basic information (e.g., personal code, gender, age),
3. Dietary survey data,
4. Blood biochemistry test results (Hemoglobin test results).

Exclusion criteria: Participants were excluded if:

1. Key variables were missing (e.g., personal code, gender, or age),
2. Daily energy intake was deemed unreasonable (defined as < 800 kcal or > 5000 kcal).

**Fig. 1:**
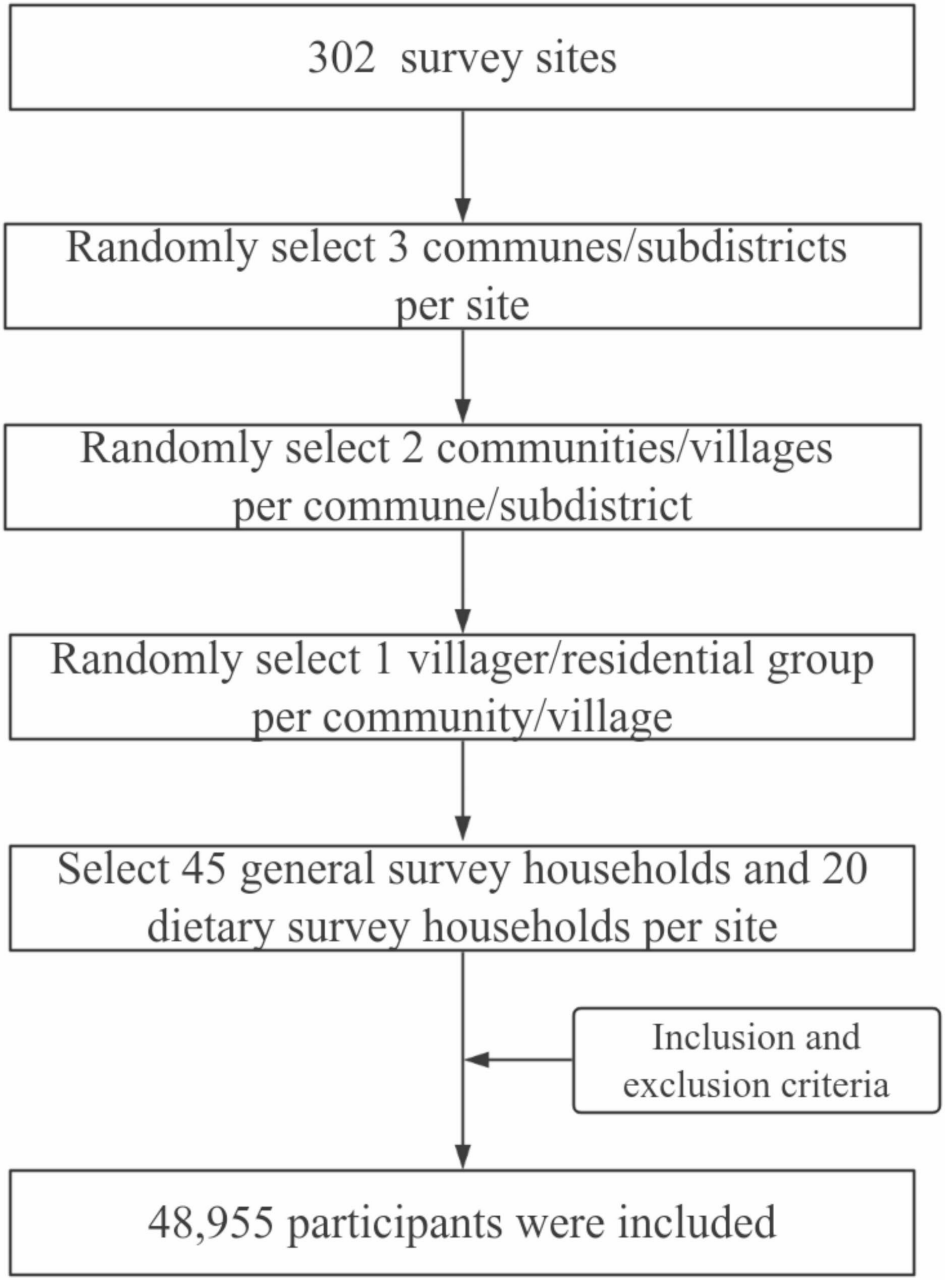
The flowchart of study design

After applying these criteria, 48,955 participants aged ≥ 60 years were included in the final analysis. A flowchart of study design was provided Fig. 1.

This study was approved by the ethical committee of the National Institute for Nutrition and Health of the Chinese Center for Disease Control and Prevention. The ethical approval number was 201519-B. All participants provided informed consent before the study.

This cross-sectional study was conducted in accordance with the STROBE (Strengthening the Reporting of Observational Studies in Epidemiology) guidelines for cross-sectional studies. The study design and methodology have been previously described in detail, specific research methods can be found in related study [19].

### Definition of anemia

According to the WHO standards [4], the impact of altitude on hemoglobin concentration was adjusted by formula, ΔHb = − 0.32 × (elevation in meters × 0.0033) + 0.22 × (elevation in meters × 0.0033)^2^, elevation-adjusted hemoglobin concentrations was used to define anemia (g/L), the specific judgment criteria were presented in Table 1.

**Table 1:** Hemoglobin concentrations (g/L)^1^ used to define anemia according to WHO criteria

	mild anemia	moderate anemia	severe anemia
Male	110–129	80–109	< 80
Female	110–119	80–109	< 80

^1^ g/L: g (grams), L (liters)

### Dietary survey and biochemical testing methods

All dietary information was collected using the food frequency method within the past year by professionally trained investigators. The food frequency questionnaire surveyed 12 major categories of food (including staple foods, legumes, vegetables, fungi and algae, fruits and dairy products, meat, aquatic products, eggs, snacks, beverages, and alcohol), and a total of 68 food groups were included. For each type of food, according to the frequency of consumption by the individuals, the frequency of consumption was filled in a column selected from “times/day”, “times/week”, “times/month”, and “times/year”. Then, according to the requirements of each food type (raw weight, dry weight, cooked weight, edible part weight, etc.), the average consumption of each corresponding frequency was recorded. And data on variables, such as gender, age, living area, degree of education, marital status, career and alcohol drinking, were collected by the China Center for Disease Control and Prevention (the China CDC) project group.

In this study, fasting venous blood samples of participants were obtained for hemoglobin detection, and the hemoglobin concentration was tested using the ferrocyanide method.

To ensure the quality of surveillance, a quality control program for the 2015 CACDNS was formulated and implemented. In the monitoring process, four unified methods were implemented, unified programs, unified manuals and questionnaires, unified training and assessment, unified equipment and reagents, and unified data entry and cleaning. All investigators and laboratory personnel were trained, practiced and assessed uniformly.

### Dietary pattern analysis

A total of 16 food groups were finally included for factor analysis to explore the dietary patterns (DPs), including rice flour and its products (mainly including rice and wheat and their products); other cereals and products (mainly including corn and its products, buckwheat, millet, etc.); fried dough products (mainly deep-fried dough sticks, fried cakes, dough balls, etc.); potatoes and products (mainly potatoes, sweet potatoes and taro, etc.); soybeans and products (such as tofu, soy milk, yuba, etc.); miscellaneous beans (such as mung beans, red beans, kidney beans, etc.); fresh vegetables; fresh fruits; fungi and algae (mainly including mushrooms, agaric, kelp, etc.); nut and products; livestock and poultry meat and products; processed products (sausages, ham sausages, luncheon meats, salted eggs, preserved eggs etc.); aquatic products (including fish, shrimp, crab and shellfish ); milk and products; eggs and products; cakes and snacks.

The average food intake of each subject was calculated based on the frequency of food intake and the average intake per time, obtained in the food frequency questionnaire. Dietary energy intake was calculated according to the Chinese Food Composition Tables [20, 21].

Principal component analysis (PCA) with VARIMAX rotation was used, and exploratory factor analysis was conducted to extract DPs. All the necessary prerequisites of PCA including the Kaiser-Meyer-Olkin (KMO) measure and the significant Bartlett’s test of sphericity (*p* < 0.001) were met. The number of extracted factors was decided by eigenvalues greater than 1, the scree plot, and the interpretability [22, 23]. The food groups contained in each dietary pattern were determined based on the absolute value of the factor load over 0.20 [24].

Factor scores for each food group were calculated by summing up intakes of food weighted by their factor loadings. The largest score showed that the diet of participants tended to correspond with the DPs. All individuals were divided into five groups according to the quintile of each DP score (Q1: 0–19% of the factor score, Q2: 20-39% of the factor score, Q3: 40-59% of the factor score, Q4: 60-79% of the factor score, Q5: 80-100% of the factor score).

### Assessment of other variables

The living area were divided into two groups based on urban and rural areas, and three groups based on regions: eastern region (including Beijing, Tianjin, Liaoning, Shandong, Hebei, Jiangsu, Zhejiang, Shanghai, Fujian, Guangdong, Hainan), central region (including Jilin, Heilongjiang, Shanxi, Henan, Anhui, Jiangxi, Hunan and Hubei), and western region (including Inner Mongolia, Ningxia, Gansu, Qinghai, Xinjiang, Tibet, Guizhou, Shanxi, Chongqing, Sichuan, Yunnan and Guangxi).

In this study, we divided all the participants into six age groups (including 60–64, 65–69, 70–74, 75–79, 80–84 and ≥ 85) [4]. The research participants were divided into three groups (primary and lower, secondary, college or above) based on their education level and two groups(unmarried, married) based on their marital status. Alcohol drinking status was divided into two groups (drinking and never).

The research participants were divided into four categories based on their occupational nature, manual laborers (including production, transportation, service personnel, household activities, etc.), mental worker (including personnel of enterprises and institutions, professional technical personnel, etc.), the retired (refers to those who have left their job position in accordance with national regulations and have not engaged in a fixed occupation again), and other workers ( other employees who are unable to classify).

### Statistical analysis

Data were analyzed by software SAS 9.4 software (SAS Institute Inc., Cary, NC, USA), R software (Version 4.3.1) and SPSS 22.0 software (SPSS Inc., Chicago, IL, USA). R software was used to drawing, SPSS 22.0 software was used to perform the Bartlett’s test, and the rest of the calculations were done by SAS 9.4. The categorical data was collected as numbers (percentage) and the Chi-square test was conducted. The associations between the quintile of NP and anemia were assessed using multivariate logistic regression analysis and trend testing, adjusted for gender, age, place of residence, regional distribution, marital status, educational level, occupation, alcohol consumption, and energy intake. The alpha level was set at 0.05.

## Results

### Characteristics of participants

A total of 48,955 elderly individuals aged 60 years and above in China were included in this study, including 24,554 males (50.15%) and 24,401 females (49.85%). The overall prevalence of anemia among all participants was 9.02% ( 5.35% with mild anemia, 3.29% with moderate anemia, and 0.38% with severe anemia). The anemia status of the survey participants under different demographic characteristics was shown in Table 2.

**Table 2:** Characteristics of the basic information [n, %]

Characteristics	no anemia	anemia				P*
		total	mild anemia	moderate anemia	severe anemia	
Gender						
Male	22,261(45.47)	2293(4.68)	1619(3.31)	603(1.23)	71(0.15)	< 0.0001
Female	22,277(45.51)	2124(4.34)	1001(2.04)	1006(2.05)	117(0.24)	
Age						
60-	18,015(36.79)	1488(3.04)	846(1.73)	558(1.14)	66(0.13)	< 0.0001
65-	12,781(26.10)	1179(2.41)	683(1.39)	449(0.92)	47(0.10)	
70-	7398(15.11)	783(1.60)	450(0.92)	297(0.61)	36(0.07)	
75-	4071(8.31)	574(1.17)	370(0.76)	176(0.36)	28(0.06)	
80-	1700(3.47)	303(0.62)	192(0.39)	100(0.20)	11(0.02)	
85-	573(1.17)	108(0.22)	79(0.16)	29(0.06)	0(0.00)	
Place of residence						
Urban	19,289(39.70)	1487(3.06)	924(1.09)	513(1.06)	50(0.10)	< 0.0001
Rural	24,910(51.26)	2907(5.98)	1690(3.48)	1081(2.22)	136(0.28)	
Region						
Eastern region	17,236(35.21)	1516(3.10)	895(1.83)	570(1.16)	51(0.10)	< 0.0001
Central region	13,522(27.62)	1288(2.63)	836(1.71)	387(0.79)	65(0.13)	
Western region	13,780(28.15)	1613(3.29)	889(1.82)	652(1.33)	72(0.15)	
Education level						
Primary and lower	30,468(62.24)	3423(6.99)	1997(4.08)	1280(2.61)	146(0.30)	< 0.0001
Secondary	12,499(25.53)	1002(2.05)	631(1.29)	332(0.68)	39(0.08)	
College or above	1466(2.99)	97(0.20)	74(0.15)	22(0.04)	1(0.00)	
Marital status						
Unmarried	371(0.76)	42(0.09)	29(0.06)	12(0.02)	1(0.00)	< 0.0001
Married	44,167(90.22)	4375(8.94)	2591(5.29)	1597(3.26)	187(0.38)	
Occupation						
Manual workers	28,899(59.03)	3192(6.52)	1820(3.72)	1225(2.50)	147(0.30)	< 0.0001
Mental workers	1235(2.52)	74(0.15)	56(0.11)	17(0.03)	1(0.00)	
Retirees	10,017(20.46)	689(1.41)	451(0.92)	213(0.44)	25(0.05)	
Other occupations	4387(8.96)	462(0.94)	293(0.60)	154(0.31)	15(0.03)	
Alcohol consumption						
No drinking	14,408(29.43)	1215(2.48)	809(1.65)	368(0.75)	38(0.08)	< 0.0001
Drinking	30,129(61.55)	3202(6.54)	1811(3.70)	1241(2.54)	150(0.31)	

*Chi-square test was applied

### The distribution of dietary patterns among elderly aged 60 and above in China

**Fig. 2:**
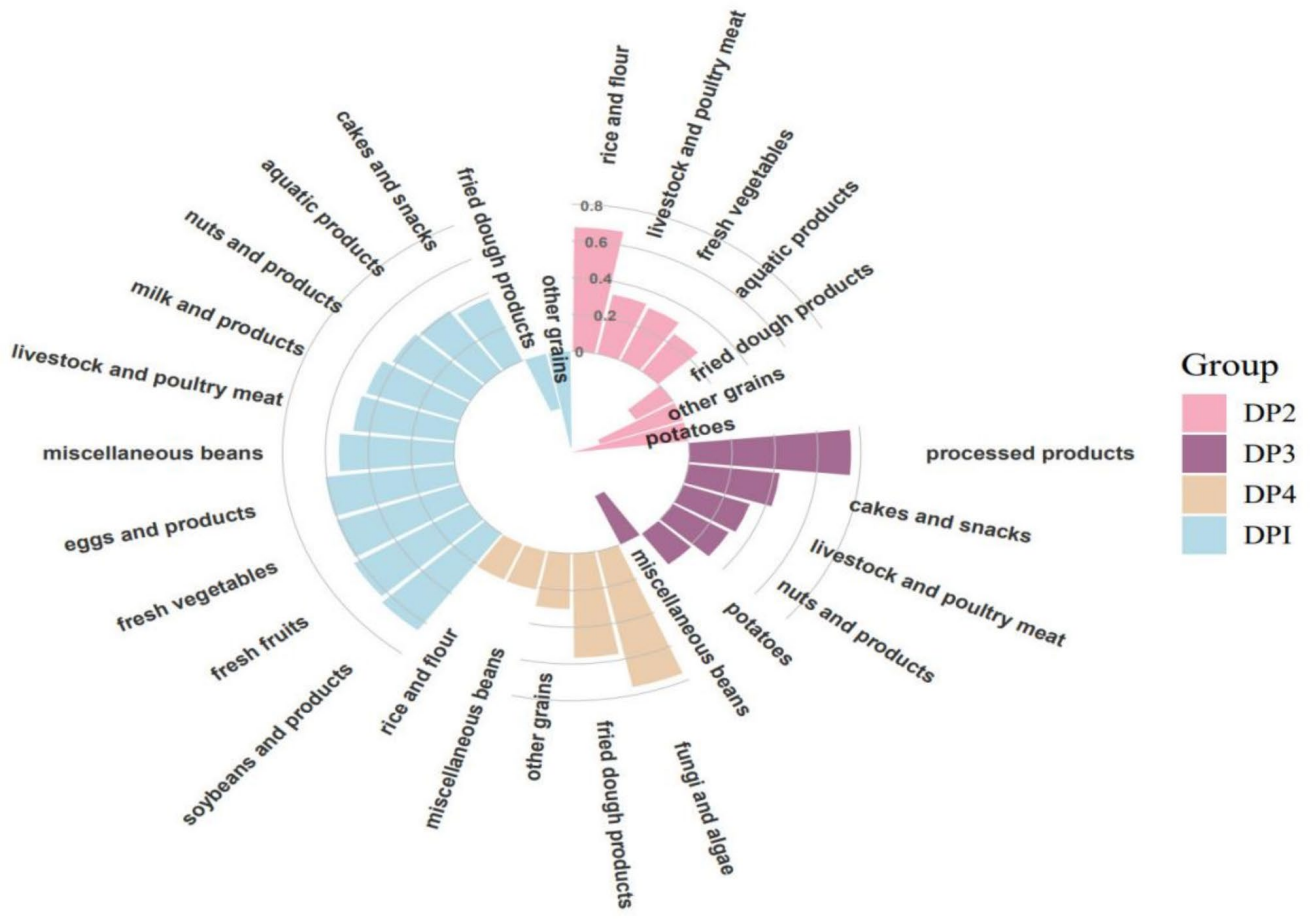
Factor loading of different dietary patterns in older adults aged over 60 years in China

Factor analysis identified four mutually exclusive dietary patterns that together accounted for 43.17% of the total variation. The four DPs could explain 22.41%, 7.82%, 6.56% and 6.38% of the total variation, respectively. As shown in Fig. 2, Dietary Pattern 1 (DP1) was characterized by high factor loadings from soybeans and products, fresh fruits, eggs and products, fresh vegetables, miscellaneous beans, livestock and poultry meat, milk and products, nuts and products, aquatic products, and cakes and snacks, and was low factor loadings from fried dough products and other grains; DP2 was rich in rice and flour and their products, fresh vegetables, livestock and poultry meat, aquatic products, and was low in fried dough products, other grains, and potatoes, Dietary Pattern 3 (DP3) included processed products, cakes and snacks, livestock and poultry meat, nuts and products, potatoes, and was low in miscellaneous beans, and DP4 was rich in fungi and algae, fried dough products, other grains, miscellaneous beans and rice and flour and their products.

The results of the distribution of four dietary patterns were presented in Table 3. Among older adults aged 60 and above in China, DP2 was the main dietary pattern, and it accounted for the highest percentage of older adults of different genders, ages, places of residence. However, the distribution of the four dietary patterns were varied somewhat across different demographic characteristics. DP2 and DP4 dominated in males, but the DP2 and DP1 dominated in females. Among the different age groups, the 80-year-old age group was dominated by DP2 and DP1, and the remaining age groups were dominated by DP2 and DP4. In urban, the main dietary pattern was DP1, however, the percentages of DP1, DP4 and the DP2 pattern were similar, at 28.04%, 26.00% and 27.65%, respectively.

**Table 3:** Distribution of four dietary patterns in older adults in China. [n, %]

Characteristics	DP1	DP2	DP3	DP4
Gender*				
Male	4946(20.14)	9456(38.51)	3022(12.31)	7130(29.04)
Female	5617(23.02)	9270(37.99)	4263(17.47)	5251(21.52)
Age				
60-	4125(21.17)	7260(37.26)	3079(15.8)	5021(25.77)
65-	3014(21.59)	5314(38.07)	2039(14.61)	3593(25.74)
70-	1774(21.68)	3224(39.41)	1138(13.91)	2045(25.00)
75-	1032(22.22)	1841(39.63)	668(14.38)	1104(23.77)
80-	470(23.46)	807(40.29)	266(13.28)	460(22.97)
85-	148(21.73)	280(41.12)	95(13.95)	158(23.20)
Place of residence*				
Urban	5826(28.04)	5402(26.00)	3803(18.3)	5745(27.65)
Rural	4635(16.66)	13,236(47.58)	3412(12.27)	6534(23.49)
Region*				
Eastern region	4605(24.56)	6495(34.64)	2756(14.70)	4896(26.11)
Central region	3233(21.83)	6067(40.97)	2159(14.58)	3351(22.63)
Western region	2725(17.70)	6164(40.04)	2370(15.40)	4134(26.86)

Chi-square test was applied; * *p* < 0.0001

The results of the distribution of four DPs were presented in Table 4. Among older adults aged 60 and above, the DP1 was adopted by a total of 10,563 ( 21.58%). The number of older adults classified as the DP2 was 18,726 (38.25%). The number with the DP3 and the DP4 were 7,285 (14.88%) and 12,381 (25.29%), respectively.

**Table 4:** Proportion of anemia in older adults in China under different dietary patterns [n (%)]

Dietary patters	No anemia	Anemia	P*
DP1	9759(92.39)	804(7.61)	< 0.0001
DP2	16,712(89.24)	2014(10.76)	
DP3	6710(92.11)	575(7.89)	
DP4	11,357(91.73)	1024(8.27)	

*Chi-square test was applied

**Fig. 3:**
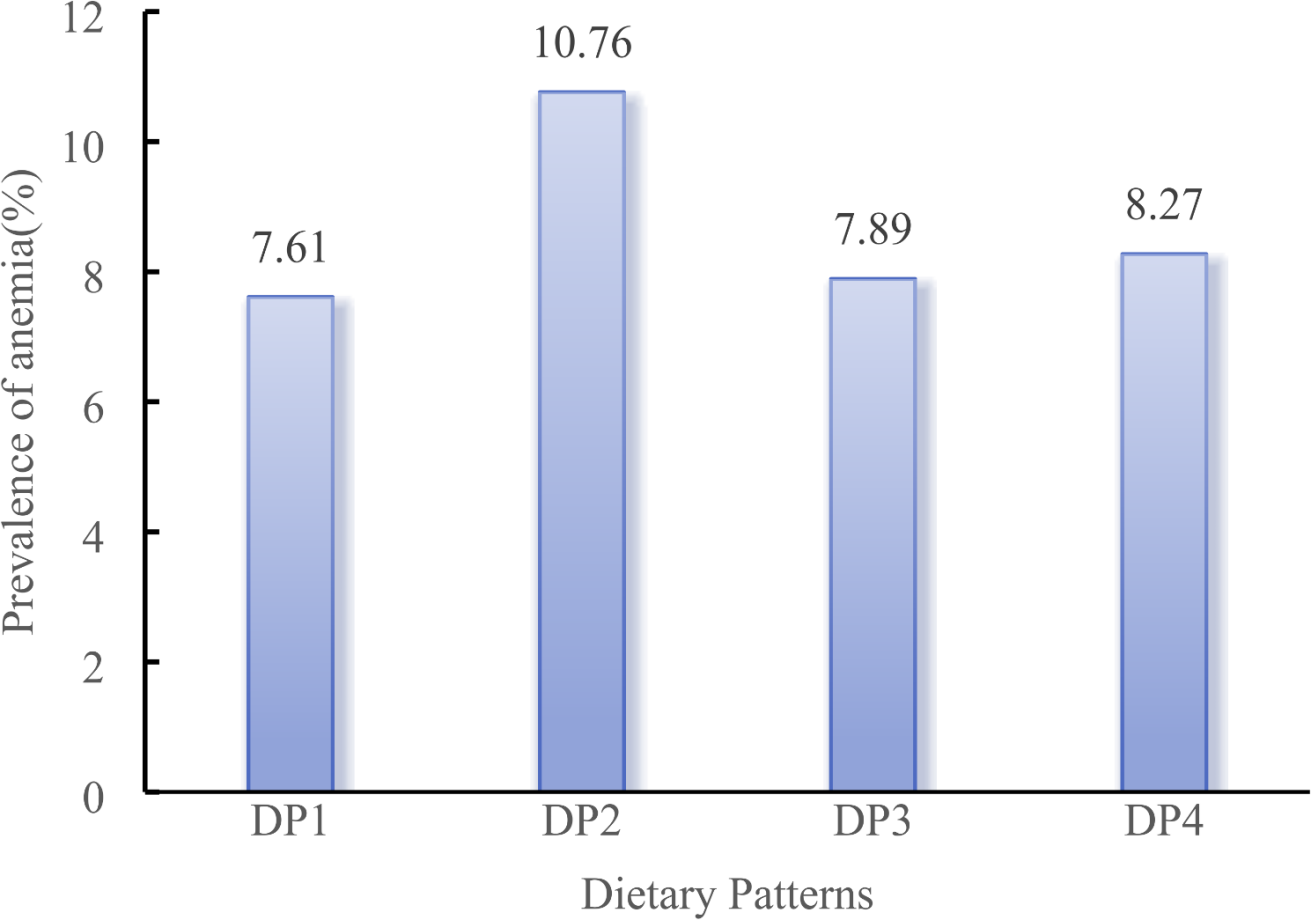
The prevalence of anemia under different dietary patterns

As show in Fig. 3, the prevalence of anemia under the DP1, DP2, DP3 and DP4 were 7.61%, 10.76%, 7.89% and 8.27%, respectively. The prevalence of anemia under the different dietary patterns was different (*P* < 0.0001), DP2 exhibited the highest prevalence of anemia.

### Associations between dietary patterns and anemia among elderly adults aged 60 and above in China

The results of the association between DPs and anemia were shown in Table 5. After adjusting for gender, age, place of residence, regional distribution, marital status, educational level, occupation, alcohol consumption, and energy intake, compared with Q1, there was an increased prevalence of anemia (OR = 1.412, 95%CI: 1.273–1.567) among individuals of the Q5 level following DP 2. And the trend test results showed that DP2 score was associated with higher prevalence of anemia (*p* for trend < 0.0001), this suggested that the closer the dietary pattern of older adults was to DP2, the higher associated with the prevalence of anemia.

There was a decreased prevalence of anemia among individuals following DP4, with a factor score in the fifth quantile compared to the first quantile. Dietary pattern 4 was linked to lower prevalence of anemia (95%CI: 0.674–0.874), and was a decrease prevalence of anemia between DP4 score and anemia (*P* for trend < 0.0001). This suggested that the closer the dietary pattern was to DP4, the lower associated with the prevalence of anemia in older adults.

**Table 5:** The associations between dietary patterns with anemia by logistic regression

	OR(95%CI)	*P*	*P* for trend
DP1			
Q1	ref		0.4191
Q2	1.078(0.963 ~ 1206)	0.1905	
Q3	1.100(0.967 ~ 1.252)	0.1470	
Q4	1.127(0.996 ~ 1276)	0.0586	
Q5	0.955(0.864 ~ 1.078)	0.4540	
DP2			
Q1	ref		< 0.0001
Q2	1.143(1.028 ~ 1.277)	0.0134	
Q3	1.202(1.082 ~ 1.377)	0.0006	
Q4	1.278(1.150 ~ 1.419)	< 0.0001	
Q5	1.412(1.273 ~ 1.567)	< 0.0001	
DP3			
Q1	ref		0.5292
Q2	1.042(0.945 ~ 1.149)	0.4062	
Q3	0.970(0.876 ~ 1.073)	0.5527	
Q4	1.047(0.944 ~ 1.162)	0.3834	
Q5	1.040(0.935 ~ 1.157)	0.4732	
DP4			
Q1	ref		< 0.0001
Q2	0.852(0.773 ~ 0.938)	0.0011	
Q3	0.808(0.729 ~ 0.895)	< 0.0001	
Q4	0.814(0.726 ~ 0.913)	0.0004	
Q5	0.768(0.674 ~ 0.874)	< 0.0001	

*The multivariate logistic regression model was adjusted for gender, age, Place of residence, regional distribution, marital status, educational level, occupation, alcohol consumption, and energy intake. OR: odds ratio, CI: confidence interval

## Discussion

The pathogenesis of anemia in older adults is complex, with chronic disease and nutritional anemia being the two main causative factors [25]. The normal function of the hematopoietic system in older adults population is directly or indirectly affected by chronic non-communicable diseases, such as kidney disease, infection, and tumor through different pathological mechanisms, such as chronic inflammation, chronic blood loss, and renal function decline. Nutritional anemia is mainly attributed to the deficiency of hematopoietic-related nutrients, such as iron, vitamin B12, folic acid, and nutritional metabolism disorders, and dietary iron deficiency remains the leading cause of nutritional anemia worldwide.

With advancing age and the declining body function, older adults may be at risk for anemia due to a variety of factors, including but not limited to inadequate dietary intake, absorption disorders, medication interference, and increased nutrient consumption caused by chronic diseases [26–31]. It is worth noting that diet plays a crucial role in the health of older adults. Anemia caused by dietary factors is relatively clear, which makes prevention and treatment more direct and effective, diet is one of the key modifiable factors for treating anemia [32, 33]. Targeted improvement of the nutritional status of older adults can not only reduce the prevalence of anemia, but also have a positive effect on improving overall health and preventing other diseases of older adults.

According to the WHO, the anemia prevalence higher than 40% is considered a serious public health problem; 20.0%~39.9% is a moderate public health problem; 5.0%~19.9% is a mild level; and the prevalence of anemia less than 4.9% is considered that anemia is not a typical public health problem [34].

This study revealed that the prevalence of anemia among older adults aged 60 and above in China was 9.02%. While this may be considered a relatively mild public health problem, its development trend is nevertheless worthy of attention. Related studies have shown that the prevalence of anemia increases with age. In this context, one of the main goals of China’s National Nutrition Plan (2017–2030) is to reduce the anemia rate of older adults [35]. The improvement of anemia among older adults in China is not only related to individual health and well-being, but also an important part of promoting the process of healthy aging of society.

Previous nutritional epidemiological analyses have focused on the association of individual nutrients or foods with disease. Some scholars have suggested that iron is found in large quantities in animal food, especially liver, and the occurrence of anemia can effectively prevent [36]. Some scholars have also found that grains increase the risk of anemia [37]. But the complex interaction between food and nutrients is ignored, and dietary intake, food, nutrients and the synergistic and antagonistic effects between them are taken into account by dietary patterns, and the relationship between diet and disease is more comprehensively and realistically responded [38].

In this study, four mutually exclusive dietary patterns were found among older adults over 60 years old. DP1 was characterized by high intake of soybeans and products, fresh fruits, eggs and products, fresh vegetables, miscellaneous beans, livestock and poultry meat, milk and products, nuts and products, aquatic products, and cakes and snacks; DP2 was rich in rice and flour and their products, fresh vegetables, livestock and poultry meat, aquatic products, DP3 included processed products, cakes and snacks, livestock and poultry meat, nuts and products, potatoes, and DP4 was rich in fungi and algae, fried dough products, other grains, miscellaneous beans and rice and flour and their products.

And our study found an increased prevalence of anemia among individuals following DP2, while a decreased prevalence was observed among those following DP4. However, no statistically significant association was observed between DP1 and DP3 and the prevalence of anemia.

The results of this study showed that DP2, characterized by high intake of rice and flour and their products, fresh vegetables, livestock and poultry meat, aquatic product, was associated with higher prevalence of anemia. Traditional dietary pattern (high intake of rice, pork and vegetables) was positively associated with anemia have been shown in similar studies [39]. Rice and flour are rich in non-heme iron and phytic acid, and the bioavailability of non-heme iron is low. Related studies have shown that the absorption efficiency of non-heme iron is about 10%, heme iron is about 25%. The absorption rate of hemoglobin iron is 2.5 times higher than that of non-heme iron. At the same time, in the gastrointestinal tract, insoluble aggregates are formed by the combination of phytic acid and iron. These aggregates are difficult to be absorbed in the physiological pH environment of the small intestine, thus hindering the absorption of iron [40–42]. Our study found that DP2 was the main dietary pattern in China, which was consistent with related studies. Given that rice and other grains are widely consumed as staple foods in China, this dietary habit may be one of the reasons for the high prevalence of anemia [43, 44]. Fresh vegetables supply a large amounts of non-heme iron, Vitamin B6, and folate. However, the absorption efficiency of non-heme iron was lower. And vegetables also contain phytate, which can reduces the absorption of non-heme iron [45]. Although livestock and poultry meat contain heme iron, it was associated with an increased prevalence of anemia. The possible reasons were as follows: Firstly, although meat from livestock and poultry was included, the intake was relatively low. Secondly, the large amounts of phytate in rice and flour and fresh vegetables can reduces heme iron bioavailability. The nutritional value of aquatic products varies significantly. For instance, small pelagics, crabs and crayfish, turtle and canned fish, are good sources of calcium, bivalves and gastropods and small pelagics are important sources of iron [46]. And small fish are often consumed whole, including the head, flesh, and organs, making them an excellent source of bioavailable heme iron. In contrast, large fish, which are typically consumed as fillets or portions, do not exhibit the same association with iron provision [47]. Therefore, the selection of aquatic products is crucial for preventing anemia in the older adults.

Focusing exclusively on the consumption of a single food group or nutrient may not sufficiently or comprehensively achieve the objective of disease prevention, particularly in the elderly population. The dietary requirements of older adults should consider the synergistic interactions among diverse foods and nutrients, as the absorption and metabolism of various dietary components may influence one another, leading to complex cumulative effects. Consequently, when designing dietary interventions for older adults, it is imperative not only to ensure dietary diversity and appropriate nutrient intake, but also to emphasize the strategic combination of foods. Achieving a nutritionally balanced diet is critical to preventing both deficiencies and excessive intake of nutrients. Balanced diet should not only meet the nutrient requirements, but also ensure adequate bioavailability to fulfill their needs. A study of the association between dietary patterns and anemia showed: dietary patterns with high intakes of eggs, meat, organ meats, rice or flour products, fried foods, sugary beverages, and processed foods significantly increased the risk of anemia, and was associated with decreased hemoglobin, hematocrit, and red blood cells. Although this dietary pattern was high in meat and organ meats, the presence of certain other food groups (such as eggs and rice), in this dietary pattern may interfere with iron absorption or bioavailability [48].

DP4, characterized by rich in fungi and algae, fried dough products, other grains, miscellaneous beans and rice and flour and their products, was linked to lower prevalence of anemia. In countries like China, Japan, and Korea, fungi and algae are commonly incorporated into daily diets for their distinctive flavors and health benefits [49]. And this DP aligns with a study conducted in Japan, which demonstrated that a dietary pattern rich in vegetables, mushrooms, seaweed, and tofu was associated with a lower prevalence of anemia. Specifically, individuals in the highest quartile of this dietary pattern had a risk of 0.829 (95% CI: 0.696 ~ 0.986, *P* = 0.035) compared to those in the lowest quartile [50]. This may be due to the fact that DP4 contains no-heme iron and ascorbic acid from fungi and algae, and miscellaneous beans, although the absorption rate of non-heme iron is lower than that of heme iron, bioavailability of non-heme iron can be increased by ascorbic acid [51]. At the same time, related research shows that seaweed is important sources of iron, seaweed contains 8 mg/100 g, which is double the amount contained by pelagics, bivalves and gastropods [46]. And related research shows that the number of human red blood cells can be increased by Auricularia auricula (wood ear mushroom, a dark-colored, edible fungus commonly used in Asian cuisines for its crunchy texture and nutritional benefits). After taking Auricularia auricula for 30 days, the content of red blood cells and hemoglobin in blood was increased. The reason may be that ions and polysaccharide macromolecules in Auricularia auricula work together to supplement proteins and trace elements required for red blood cell synthesis, and can also repair damaged red blood cells [52, 53].

Although DP1 and DP3 have not been found to be associated with the prevalence of anemia, there have been related studies on the foods contained in these patterns. DP1, characterized by high factor loadings from soybeans and products, fresh fruits, fresh vegetables and eggs and products. Although studies have shown that soybeans can improve the prevalence of anemia [53, 54], the phytates and tannins in plants can chelate non-heme iron [55]. DP3 characterized by being rich in processed products, cakes and snacks, livestock and poultry meat. Study found that the fast-food pattern (high intake of sweets, fast food, etc.) was associated with higher prevalence of anemia [32]. This may be due to the low iron content in fast food, which is about 4% of the recommended amount of iron [56].

The associations found in this study may be influenced by several potential confounding factors that were not adjusted for in the statistical model. Specifically, chronic diseases, absorption disorders (such as gastrointestinal problems), drug interference, and acute diseases causing inflammation could mediate the relationship between the dietary patterns and anemia. Moreover, most of the elderly adults take drugs due to various underlying diseases, drug interference is another important variable that could impact the results. In addition, there are other important prognostic factors that may influence anemia, including demographic factors like lifestyle factors such as physical activity, smoking, drinking. These variables could have confounded the observed relationships, however, due to limitations in the available data, these variables could not be included in the analysis. To provide a more comprehensive understanding of the factors associated with anemia, further studies incorporating these factors are need to collect and incorporate these important variables.

This research was based on nationwide research, scientific design, large sample size, wide coverage and good representation were the advantages of this research. However, limitations were also present in this study. The main limitation is that this study is a cross-sectional study, which makes it impossible for us to make causal inferences. Our research did not include oil and condiments such as salt, so the relationship between them and anemia in older adults in our country cannot be obtained, and the energy intake is underestimated. And recall bias existed in our study, as dietary data were obtained mainly by the respondents’ recalls. To reduce recall bias, all data were collected by uniformly trained and experienced investigators. And more large prospective cohort studies are needed to explore the relationship between dietary patterns and anemia.

## Conclusions

Heme iron in livestock and poultry meat plays a crucial role in the prevention of anemia. However, it is important to consider the impact of other nutrients, such as phytic acid and vitamin C, on the bioavailability of heme iron. While iron from fungi and algae is non-heme iron, these sources contain relatively high levels of non-heme iron, and the complex interaction of nutrients in these foods can enhance the absorption of non-heme iron. Therefore, the potential of fungi and algae as food sources for preventing and improving anemia should not be overlooked. From a public policy perspective, addressing anemia among older adults in China requires more focus on comprehensive nutritional interventions. Targeted dietary measures should prioritize a balanced intake of various nutrients rather than focusing on a single nutrient or food source. And it is recommended that elderly individuals increase their intake of fungi and algae to prevent anemia.

## Data Availability

The datasets used and/or analysed during the current study are not publicly available according to the policy of the National Institute for Nutrition and Health, Chinese Center for Disease Control and Prevention, but are available from the corresponding on reasonable request.

## Abbreviations

WHO: World Health Organization
HF: Heart Failure
CAD: Coronary Artery Disease
2015 CACDNS: 2015 China Adults Chronic Diseases and Nutrition Surveillance
China CDC: China Center for Disease Control and Prevention
DP: Dietary Pattern
APC: Principal component analysis
KMO: Kaiser-Meyer-Olkin
OR: Odds Ratio
95%CI: 95% Confidence Interval
